# Supporting the mental health of adolescent mothers in Kenya and Mozambique: pilot protocol for the Thriving Mamas programme

**DOI:** 10.1186/s40814-025-01617-5

**Published:** 2025-06-19

**Authors:** Tatiana Taylor Salisbury, Maria Suzana Bata Maguele, Fernando Chissale, Málica de Melo, Margrette Hanselmann, Kethakie Lamahewa, Evaline Lang’at, Flávio Mandlate, Lucy Nyaga, Nadine Seward, Marleen Temmerman

**Affiliations:** 1 Health Service and Population Research Department, Institute of Psychiatry, Psychology and Neuroscience, King’s College London, Denmark Hill, London, SE5 8AF UK; 2 International Centre for Reproductive Health Mozambique, Rua das Flores 34, Impasse 1085/87, Maputo, Mozambique; 3 Centre of Excellence in Women and Child Health, Aga Khan University – East Africa, 3rd Parklands Avenue, PO Box 30270, Nairobi, 00100 Kenya; 4 Department of Mental Health, Ministry of Health, Avenida Eduardo Mondlane 1008, C.P. 264, Maputo, Mozambique; 5 Faculty of Medicine, Eduardo Mondlane University, Avenida Salvador Allende nº. 702, C. Postal 257, Maputo, Mozambique; 6 Centre for Clinical Brain Sciences, University of Edinburgh, 49 Little France Crescent, Edinburgh, EH16 4SB UK

**Keywords:** Perinatal mental health, Adolescent mental health, Intervention

## Abstract

**Background:**

Poor mental health among adolescent girls during pregnancy and the year after birth (the perinatal period) has been found to have a significant detrimental effect on girls and their children. The Innovative approaches to adolescent perinatal wellbeing (INSPIRE) project co-designed an intervention (Thriving Mamas) to improve adolescent perinatal mental health in Kenya and Mozambique. The aim of the current study is to pilot test the co-designed intervention to understand how it can be adapted for further testing or scale-up.

**Methods:**

A mixed-method, pilot cluster-randomised, effectiveness-implementation Hybrid Type II trial will be conducted among 80 adolescent girls (aged 15–19 years) attending health facilities in Kenya and Mozambique. Girls attending health facilities randomised to the intervention arm will be recruited to receive the intervention in addition to usual care. Girls attending control arm facilities will receive usual care only. Implementation data on feasibility, acceptability, appropriateness, and fidelity of intervention training and delivery will be collected from providers and adolescents. Additional data on provider knowledge, attitudes, and competency will be collected pre- and post-training. Adolescent girls will provide assessments of depression, anxiety, quality of life, social support, parenting competency, and health behaviours before, during, and after delivery of the intervention. At the end of the study, qualitative interviews will be conducted to collect further data on perceptions of the intervention, its implementation, and its impact. Implementation data will be analysed to determine its potential for delivery and success within each context. Individual outcome data analyses will be reported to gain a better understanding of the initial impact of the intervention.

**Discussion:**

The Thriving Mamas intervention addresses the challenges to maintaining positive mental health during the perinatal period. It was co-designed with adolescents and their communities to address local priorities and needs. The results of this study will provide information on its potential for sustainable implementation as well as initial data on its impact on mental health, parenting, and health behaviour outcomes. These results will inform the further refinement of the intervention and implementation strategies as well as the design of a full trial to test their effectiveness.

**Trial registration:**

Ethical approval for this feasibility study has been obtained in Kenya (Aga Khan University Institutional Scientific and Ethics Review Committee, ref: 2023/ISERC-23 (v2)), Mozambique ( Tete Inter-Institutional Health Bioethics Committee, ref: 24/CIBST/23), and the UK (Psychiatry, Nursing and Midwifery Research Ethics Committee, King’s College London, ref: HR/DP-22/23–37,125). It was registered at Clinicaltrials.gov (NCT06040359) on September 15, 2023 (https://clinicaltrials.gov/study/NCT06040359?intr=Thriving%20mamas&rank=1 ), prior to the beginning of the study.

## Background

Mental wellbeing during the perinatal period (i.e. pregnancy and the year after childbirth) is critical due to its important influence on the physical and mental health and social outcomes of mothers and their children. Pregnant adolescents in low- and middle-income countries (LMICs) often experience a variety of adverse events (e.g. family conflict, poor social support, poor self-esteem, gender discrimination, exclusion from education, and poverty) which increase their risk for mental health problems [1]. In Kenya and Mozambique, an estimated 1 in 3 adolescents experience a mental disorder during pregnancy and the year after birth [2, 3]. Despite a clear need for support, no adolescent maternal mental health prevention interventions have yet been evaluated in sub-Saharan Africa, where the majority of the world’s adolescent mothers live [4].

The Innovative approaches to adolescent perinatal wellbeing (INSPIRE) project aims to address the implementation difficulties of mental health interventions through a blend of human-centred design [5], systems thinking [6, 7], and implementation science [8]. From October 2021 to February 2023, the research team partnered with key stakeholders (i.e. adolescent girls, their families, service providers, and community representatives) to identify priority challenges to adolescent perinatal mental health and develop an intervention to address them [9]. This protocol provides detail of the pilot evaluation of the intervention. The results will inform the further refinement of the intervention and implementation strategies as well as the design of a full trial to test their effectiveness. Through these methods, it is hoped that the resulting intervention will align with the needs, goals, and priorities of those directly and indirectly involved in its success and have increased chances of integration into existing health and community systems.

### Aim and objectives

The aim of the pilot is to refine the Thriving Mamas programme to improve its implementation and impact on the mental health and wellbeing of pregnant adolescent girls and new mothers aged 15–19 years living in Kenya and Mozambique. To achieve this goal, the study has five objectives:

1. Evaluate and understand how local contexts influence stakeholder perceptions of the acceptability, appropriateness, and feasibility of the intervention and its implementation strategies.
2. Explore the impact of the intervention on adolescent mother’s mental health, quality of life, social support, parenting, and health behaviour outcomes.
3. Compare implementation and clinical outcomes in Kenya and Mozambique.
4. Estimate the cost of delivering the intervention.
5. Refine the training materials, intervention content, and procedures for implementation.

## Methods

### Study design

An effectiveness-implementation Hybrid Type II pilot cluster-randomised control trial to concurrently assess and adapt intervention content and delivery will be carried out to address the study research questions and objectives over 12 months. Adolescent girls (aged 15–19 years) will be recruited from health and community settings in which participants randomised to receive the intervention or act as a control. A variety of information, including feasibility, acceptability, appropriateness, fidelity, health and wellbeing, service utilisation, and family planning, will be collected using both quantitative and qualitative methods before, during, and after the intervention. This feedback will be used to further refine the provider training, intervention, and implementation strategies.

### Setting

**Fig. 1:**
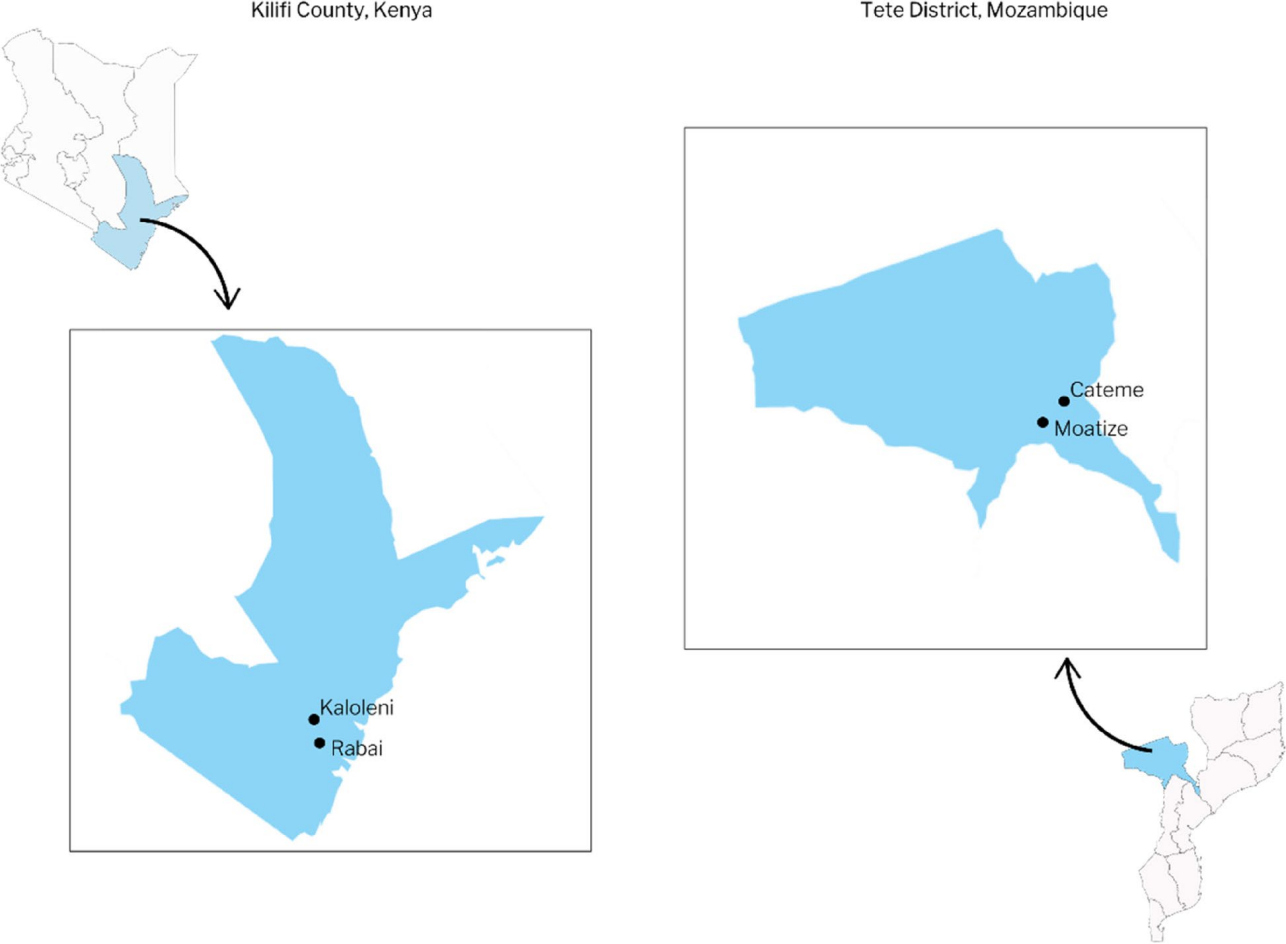
Location of Tete Province, Mozambique (right), and Kilifi County, Kenya (left)

Research will take place in health and community settings with high rates of adolescent pregnancy in Kilifi County, Kenya, and Tete Province, Mozambique (see Fig. 1). Kilifi County is located in south-west Kenya with a population of approximately 1,453,787 [10]. There are approximately 5,000 births annually.

Approximately half of these births are to women under 20 years old [11]. The research will be carried out in two sub-counties of Kilifi, Kaloleni and Rabai, which represent urban and peri-urban settings, respectively. Tete province is located in north-west Mozambique. It has a population of approximately of about 2,648,941 [12]. The estimated adolescent (aged 15–19 years) fertility rate in the province is 198.4 births per 1,000 [12, 13]. In Mozambique, the project will be conducted in Moatize City, the district capital, and Cateme, a rural area approximately 40 km from Moatize City within Tete Province.

### Participants and sampling method

The sample size for the pilot has been selected on the grounds of feasibility of recruitment and retention of participants in both study arms and was informed by local partners and stakeholders. This study aims to enrol 80 adolescent girls and 32 mentor mothers, with a loss to follow-up of 33% for both cadres of study participants. This will enable estimation of the loss of follow-up rate with a 95% CI of ± 17% for mentor mothers and ± 11% for adolescent girls [14, 15]. This, in turn, will provide us with essential information required to calculate the sample size for the main trial.

We also anticipate that our sample size will provide us with insight into whether the intervention is acceptable, appropriate, feasible, and delivered with high fidelity. If our findings suggest otherwise, this will indicate that further work is needed to adapt the intervention and selected implementation strategies, prior to implementation.

#### Mentor mothers

**Fig. 2:**
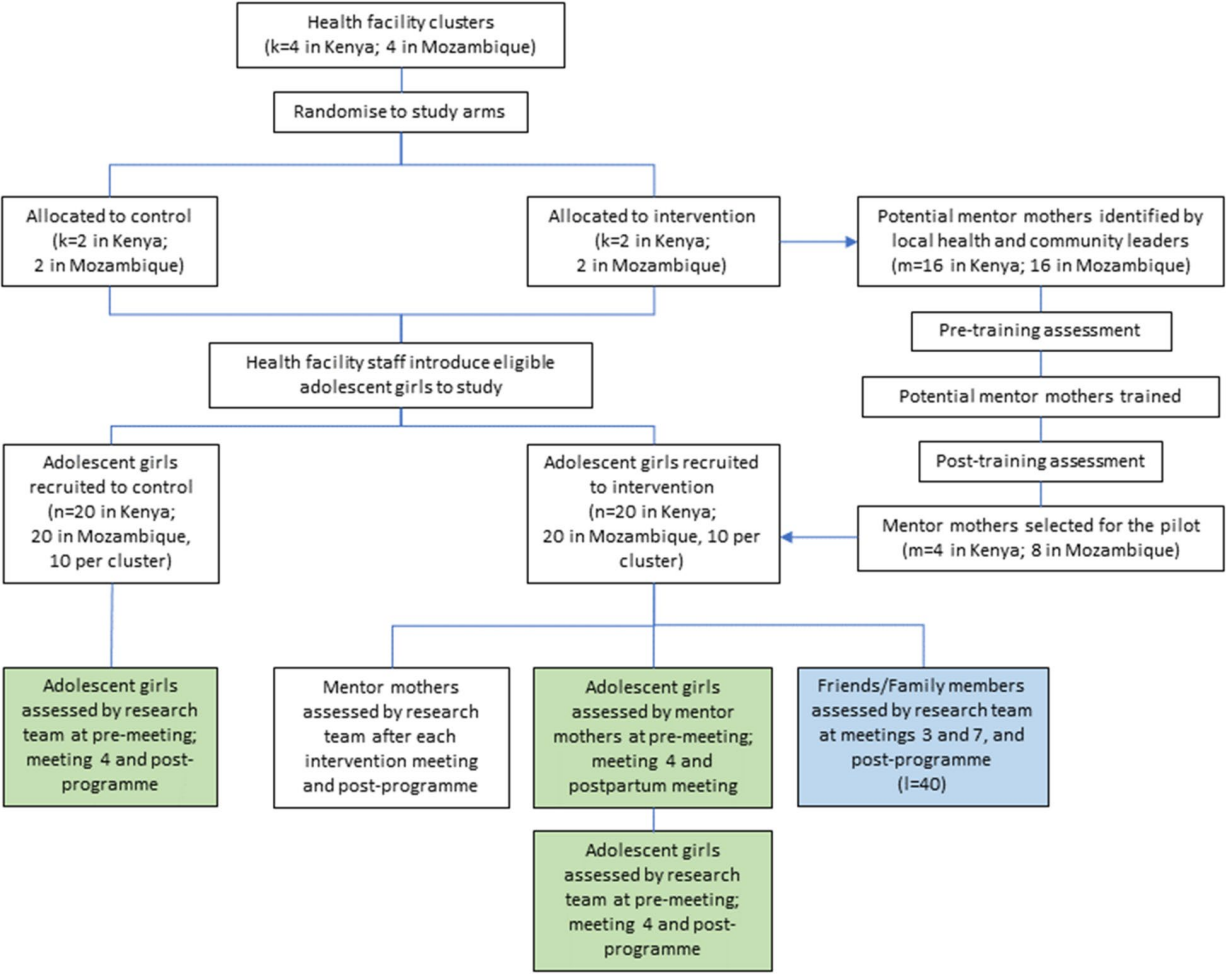
Pilot flow diagram

Up to 16 women will be recruited in each country (total of 32 women across the project) to be trained to deliver the intervention (see Fig. 2). The following inclusion criteria will be used: (a) aged 20 years or older; (b) female; (c) experience of pregnancy and/or parenting; and (d) live within the study site. Women will be excluded from participating if they are unable to attend training or deliver the intervention sessions. Following training, eight women in Mozambique and four women in Kenya will be selected to provide the intervention within the pilot study. Selection will be based on knowledge and attitude scores obtained after training.

#### Adolescent girls

Up to 40 adolescent girls will be recruited in each country (*n* = 80), through participating community services and health facilities. In Kenya, adolescents will be recruited from local dispensaries. In Mozambique, adolescents will be recruited from primary health centres. The following inclusion criteria will be used: (a) up to 28 weeks pregnant; and (b) aged 15–19 years. They will be excluded from the study if they are unable to provide informed consent or are unable to participate in the intervention, due to existing health conditions.

Adolescent girls who meet the study inclusion criteria will be given information about the study by health facility staff. Those interested in participating will be introduced to a member of the research team, who will provide them with an information sheet and consent form. Where an adolescent is aged 15–17 years, the research team also will discuss the information with their guardian during the same visit, if the guardian is in attendance. If the guardian is not present, a member of the research team will discuss the information sheet with the minor. If she indicates further interest in participating, we will explain that we also require guardian consent due to her age. Following introduction to their guardian, the study will be introduced using the information sheet and consent requested. In this situation, the minor will be required to provide their assent following guardian consent.

#### Friends/family members

Each girl randomised to receive the intervention will be given the opportunity to invite one trusted friend or family member to specific intervention sessions (*n* = 40; see description of intervention arm for details of sessions). The following inclusion criteria will be used: (a) identified by an adolescent or young woman participating in the study; (b) participation agreed by other participating girls; and (c) aged 18 years or older.

### Randomisation

Allocation to the intervention or control group will be carried out by randomisation of health facilities (clusters). This will ensure the inclusion of an urban and peri-urban site in both the intervention and control groups (i.e. stratification). In both countries, clusters will be geographically separated to reduce the potential for contamination.

### Intervention arm: the thriving Mamas programme

**Fig. 3:**
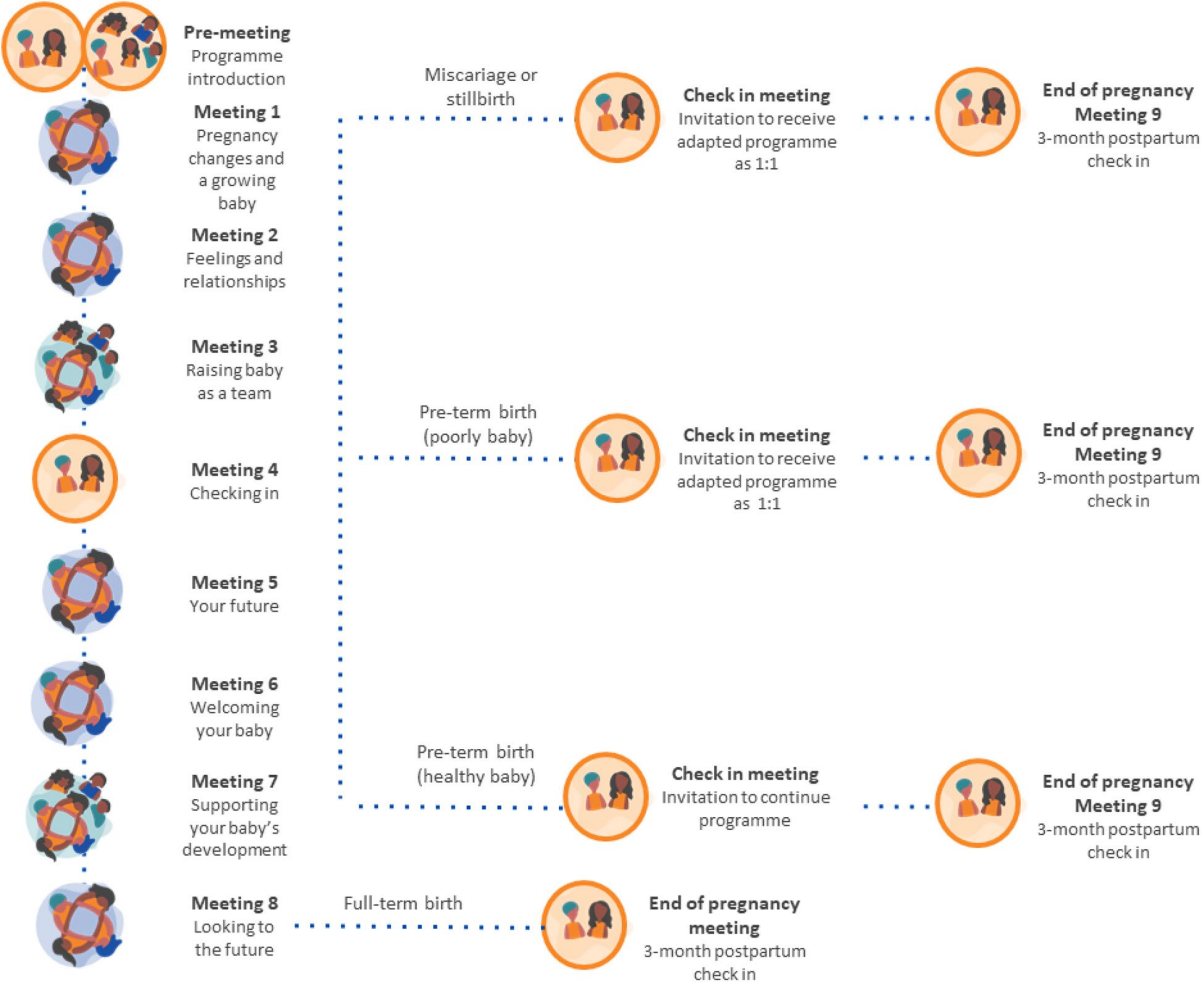
Thriving Mamas programme overview

The Thriving Mamas programme aims to support adolescent girls’ wellbeing during pregnancy and the year after birth. The programme consists of nine meetings delivered in either group (5 meetings), individual (1 meeting), or family group (2 meetings) formats over 10 weeks with an additional individual meeting 10–12 weeks postpartum (see Fig. 3). The intervention will be provided in addition to usual perinatal care. Each meeting focuses on physical and mental health, taking care of a newborn, life skills, planning for the future, and harnessing social support. Girls identified as requiring additional support will be referred to health and community-based services. The programme is facilitated by 1–2 women recruited from the local community who have been previously pregnant. Meetings will take place in a mixture of private settings in health facilities, community facilities, and participant homes. Each meeting will last between one and two hours. All mentor mothers will receive training over three to five days to deliver the programme.

### Control arm

Adolescents randomised to participate in the control arm of the trial will receive usual care provided in each country. Each visit includes care that is appropriate to the overall condition and stage of pregnancy and should include four main categories of care:

- Identification of pre-existing health conditions.
- Early detection of complications arising during pregnancy.
- Health promotion and disease prevention.
- Birth preparedness and complication planning.

In both arms, all participants will be screened for depression and anxiety during pregnancy and after participation in intervention sessions. Adolescents who screen positive for depression or anxiety will be referred for further assessment and care, where needed. Disclosure of or suspected gender-based violence will be provided with information on their rights and where to seek support based on local care provision in each country.

### Variables and data collection procedures

An overview of the assessment tools and measures used to collect the variables of interest to this study and data collection timepoints is provided in Table 1. In line with current best practice in implementation research [16], the research team will evaluate implementation variables as assessed by adolescent girls, mentor mothers, and friends/family members. This offers a 360-degree implementation evaluation, which considers needs and perspectives that typically differ between groups. Quantitative data will be collected through self-report (mentor mothers) or an interview with a member of the research team (adolescent girls, friends/family members, mentor mothers). Qualitative interviews will be conducted by one member of the research team, audio recorded, and last up to one hour.

**Table 1:** Variables of interest, assessment periods, and data collection timepoints

Variable	Measure [no. items]	Timepoint												
		Training		Intervention meetings										
		Pre	Post	Pre	1	2	3	4	5	6	7	8	9	Post
**Mentor Mother—trained to deliver the intervention**														
**Sociodemographic**														
*Age*	Sociodemographic questionnaire [7]	X												
*Area of residence (neighbourhood)*		X												
*Marital status*		X												
*Number of children*		X												
*Adolescent pregnancy*		X												
*Education status*		X												
*Occupation*		X												
**Training**														
*Knowledge*	Intervention topic quiz [16]	X	X											
*Attitudes*	Attitudes towards adolescent pregnancy [4] Social Distance Scale (SDS) [12]	X	X											
*Competency*^a^	Question on delivery of the intervention [1]		X											
*Acceptability*^a^	Acceptability of Intervention Measure (AIM) [4]		X											
*Feasibility*^a^	Feasibility of Intervention Measure (FIM) [4]		X											
*Appropriateness*^a^	Intervention Appropriateness Measure (IAM) [4]		X											
*Qualitative Interview*	Semi-structured interview		X											
**Intervention content and delivery**														
*Recruitment rate*	Programme notes			X										
*Retention rate*	Programme notes				X	X	X	X	X	X	X	X	X	
*Referral Rate*	Programme notes			X				X					X	
*Acceptability*^a^	Acceptability of Intervention Measure (AIM) [4]		X											X
*Feasibility*^a^	Feasibility of Intervention Measure (FIM) [4]		X											X
*Appropriateness*^a^	Intervention Appropriateness Measure (IAM) [4]		X											X
*Fidelity*^a^	Adolescent retention rate (fidelity of receipt)				X	X	X	X	X	X	X	X	X	
	Enhancing Assessment of Common Therapeutic Factors (ENACT) [10] (delivery fidelity; collected from adolescent girls)												X	
*Cost of intervention*	Time taken to be trained and deliver the intervention; cost of materials for delivery		X											X
*Qualitative Interview*	Semi-structured interview		X											X
**Adolescent girls**														
**Sociodemographics**														
*Age*	Sociodemographic questionnaire [12]			X										
*Neighbourhood*				X										
*Household members*				X										
*Marital status*				X										
*Parity*				X										
*Education*				X										X
*Employment*				X										X
**Health and wellbeing**														
*Depression*^a^	Patient Health Questionnaire (PHQ-9) [9]			X				X					X	
*Anxiety*^a^	Generalised Anxiety Disorder (GAD-7) [7]			X				X					X	
*Quality of life*^a^	WHO Quality of Life short version (WHOQoL-BREF) [26]			X										X
**Social support**														
*Social support*^a^	Multidimensional Scale of Perceived Social Support (MSPSS) [12]			X										X
**Parenting competency**														
*Parenting competency*^a^	Parenting Sense of Competence Scale [17]													X
**Health behaviours**														
*Perinatal appointment attendance*^a^	Information on attendance to appointments will be based on self-report by adolescent girls			X				X						X
*Referral uptake*^a^	Information on attendance to appointments will be based on self-report by adolescent girls													X
*Infant vaccination*	Health behaviours questionnaire [1]			X										X
*Breastfeeding*	Health behaviours questionnaire [1]			X										X
*Intended time to next pregnancy*^a^	Health behaviours questionnaire [2]													X
*Contraceptive use*^a^	Health behaviours questionnaire [2]													X
**Intervention content and delivery**														
*Acceptability*^a, b^	Acceptability of Intervention Measure (AIM) [4]							X						X
*Appropriateness*^a, b^	Intervention Appropriateness Measure (IAM) [4]							X						X
*Perception of intervention*^a, b^	Tailored questionnaire [6]			X	X	X	X	X	X	X	X	X	X	
*Provider Competency*^a, b^	Enhancing Assessment of Common Therapeutic Factors (ENACT) [10]												X	
*Qualitative interview*	Semi-structured interview													X
**Friends/family members**														
**Sociodemographics**														
*Age*	Sociodemographic questionnaire [7]						X				X			
*Gender*							X				X			
*Relationship to adolescent*							X				X			
*Living with adolescent*							X				X			
*Marital status*							X				X			
*Education status*							X				X			
*Employment status*							X				X			
**Intervention content and delivery**														
*Acceptability*^a^	Acceptability of Intervention Measure (AIM) [4]													X
*Appropriateness*^a^	Intervention Appropriateness Measure (IAM) [4]													X
*Perception of intervention*^a^	Tailored questionnaire [5]						X				X			
*Qualitative interview*^b^	Semi-structured interview													X

^a^*Qualitative interview topic*

^b^Completed only by adolescent girls receiving the intervention

#### Implementation variables

##### Recruitment, retention, and referral rates

Recruitment data will be collected by staff at health facilities and mentor mothers. Recruitment site staff will collect data on the number of adolescents who meet the inclusion criteria and the number who agree to be contacted by a mentor mother. Mentor mothers will collect data on the number of adolescents whom they meet with the numbers who provide consent. Reasons for not consenting will be recorded. Retention and referral rates will be extracted from mentor mother meeting records.

##### Acceptability, appropriateness, and feasibility

Acceptability, appropriateness, and feasibility of the Thriving Mothers programme and training will be assessed among mentor mothers, adolescent girls in the intervention arm, and friends/family members using the Acceptability of Intervention Measure (AIM), Intervention Appropriateness Measure (IAM) and Feasibility of Intervention Measure (FIM), respectively [17]. These brief, four-item tools were developed by implementation experts and display good psychometric properties in high-resource settings. They have been selected as they are commonly assessed in feasibility and pilot studies and, therefore, can be used to compare the intervention’s potential for scale-up with other interventions. Assessment of training will be completed by mentor mothers pre- and post-training. Assessment of the intervention will be completed pre- and post-intervention among mentor mothers and post-intervention among adolescents and friends/family members. Additional data will be obtained post-intervention through semi-structured qualitative interviews with mentor mothers, adolescents, and friends/family members.

##### Fidelity

Data on the number of sessions attended by girls and their friends/family members will be collected from mentor mothers. Attendance records completed by mentor mothers will be used to assess fidelity of intervention receipt. The extent to which the intervention was delivered as intended by the mentor mothers will be assessed by adolescent girls using the Enhancing Assessment of Common Therapeutic Factors (ENACT) tool post-intervention [18]. Qualitative interviews with mentor mothers and adolescent girls will provide a deeper understanding of these issues.

##### Operating costs

The time taken to be trained and provide the intervention as well as the costs associated with delivery (e.g. materials, transport, refreshments) will be estimated by the research team to derive operating costs for economic evaluations.

#### Mentor mothers

Information on the impact of training and delivering the intervention will be collected from mentor mothers pre- and post-training.

1. *Knowledge*: Mentor mothers’ knowledge of issues regarding pregnancy, childbirth, caregiving, mental health, and referral pathways will be assessed using a study-specific quiz based upon the intervention manual.
2. *Attitudes towards mental health*: The Social Distance Scale (SDS) was designed by Bogardus [19] to measure the level of acceptability of various types of social relationships between Americans and members of common ethnic groups [20, 21]. A modified SDS has been widely used to measure mental health-related stigma and to understand the importance of labels attached to people with former mental illnesses [20, 22]. The modified version consists of questions that represent social contact with different degrees of distance. The SDS measures the acceptability of different degrees of social distance and thus, by inference, the attitude of the respondent to the person with the condition [23, 24]. The SDS total score represents the attitude of the respondent towards the condition.
  The SDS will be used to determine the level of stigma associated with mental health among mentor mothers. As the intervention will address risk factors of poor mental health and support positive mental health among adolescents, it is important that they do not hold negative attitudes towards mental health and those living with mental health conditions. Among stigma measures, it has been shown to most strongly associate with health worker competence [25].
3. *Attitudes towards adolescent pregnancy and mental health*: Four questions were developed for the study to assess mentor mothers’ attitudes towards adolescent pregnancy.

#### Adolescent girls

A variety of variables will be collected from adolescents by mentor mothers and/or a member of the research team at pre-intervention, meeting 4 and post-intervention to investigate the impact of the intervention.

1. *Depression*: Depression is the primary outcome for our study. The Patient Health Questionnaire (PHQ-9; [26, 27]) is a self-administered screening instrument for common mental disorders. The PHQ-9 is the depression module, which scores each of the 9 DSM-IV criteria as “0” (not at all) to “3” (nearly every day). The PHQ-9 has been validated in Kenya [28] and Mozambique [29].
2. *Anxiety*: The Generalised Anxiety Disorder (GAD-7) measure is a seven-item screening tool for anxiety disorder. Each item scores a symptom of anxiety from “0” (not at all) to “3” (near every day). A GAD-7 score ≥ 10 indicates moderate to severe anxiety. The GAD-7 has been validated in Kenya [30] and Mozambique [31].
3. *Quality of life*: Quality of life will be assessed using the short version of the WHO Quality of Life brief version (WHOQOL-BREF; [32]). The WHOQOL-BREF assesses a respondent’s perceived quality of life across four domains: physical health; psychological; social relationships; and environment. Its 26 items are scored on a five-point Likert scale with higher scores indicating greater agreement. The items are allocated to each domain to provide domain scores with higher scores indicating greater quality of life in a particular domain. The WHOQOL-BREF has been used with adolescents in Kenya [33].
4. *Social support*: The Multidimensional Scale of Perceived Social Support (MSPSS) is a 12-item scale which measures social support from family, friends, and significant others [34]. Each item scores the extent to which the respondent agrees with the statements on a Likert scale with “1” (very strongly disagree) to “7” (very strongly agree). The scale has been validated in adolescents in Ghana [35].
5. *Parenting competency*: The Parenting Sense of Competency Scale is a 17-item scale to measure parents’ perceived parenting abilities [36]. The items are rated on a 6-point Likert response scale from “strongly disagree” to “strongly agree.” Overall parenting competence is derived from a total score where higher scores indicate greater perceived competence. The scale has been used to evaluate parenting competence among mothers of infants aged 2–12 months [37, 38].
6. *Service utilisation*: Antenatal appointment attendance information will be collected from adolescent girls through two questions (i.e. How many antenatal appointments were you scheduled to attend since our last meeting? How many of those appointments did you attend?). Uptake of referrals to community services will be collected from adolescent girls through three questions (i.e. Have you been referred to any community services by your intervention provider? If yes, which services have you been referred to? If yes, have you made contact with these services?).
7. *Health behaviours*: Family planning intentions will be assessed using quantitative questions regarding actual or intended contraception use and intended time to next pregnancy. Actual or intended breastfeeding and infant vaccinations will also be collected through a single question for each variable to determine the impact of the intervention on these behaviours.
8. *Provider competency*- The Mentor mothers’ competency will be assessed using the ENACT tool which was adapted to improve relevance to the study. ENACT tool measures a set of therapeutic competencies required for the effective intervention, including the delivery by the non-specialists [39].

### Data management

Paper data and documentation containing personal identifiers will be held in locked cabinets in locked offices in each implementation site. Data collected using pen and paper methods will be entered into an Excel spreadsheet independently by two members of the research team. Qualitative data from audio recorded (MPEG-1 Audio Layer 3; mp3) individual interviews will be anonymised and transcribed (Rich Text File) in the language of the interview. All electronic files will be encrypted, password-protected, and using a secure network.

### Data analyses

The primary outcomes of this pilot are the feasibility, acceptability, appropriateness, and fidelity of the Thriving Mamas programme and provider training. Recommendations for implementation-focused pilot studies suggest the use of descriptive statistics to inform a larger scale evaluation [40]. They are not designed to detect a treatment effect. Our data analysis plan is developed in line with these recommendations and informed by the CONSORT extension of randomised pilot and feasibility trials [41] and the Standards for Reporting Implementation Studies (STAaRI; [42]) guidelines.

#### Quantitative analysis

Descriptive statistics of implementation outcomes (FIM, AIM, IAM) will be calculated. These measures will be compared with different baseline characteristics (e.g. age) to develop further insight into influencing factors. Continuous outcomes (PHQ-9 and GAD-7) will be evaluated using linear regression models that adjust for clustering using a dummy variable (due to the small number of clusters), urban vs rural setting, and any obvious imbalances between treatment arms determined at baseline. Coefficients and associated 95% confidence intervals for these outcomes represent differences in means between intervention and control arms. Mean differences from baseline to the end of the intervention will be assessed for clinical outcomes (e.g. quality of life, social support, and parenting competency) in each arm. These will be discussed with local stakeholders to aid in determining clinical minimally important differences (MIDs) to inform the sample size for a full trial. The cost of training and intervention delivery will be tallied to provide a total cost of intervention delivery. Data on the costs to adolescents and friends/family members in taking part in the intervention will contribute to the assessment of the feasibility and sustainability of the intervention.

Patterns and the proportion of missing data will be investigated. If there are any marked differences in missing data between treatment arms, or if greater than 10% of the data are missing, multiple imputation using chained equations (MICE) models under the assumption that data are missing-at-random will be used for all the analyses described above. All analyses will be conducted in STATA v.17.

#### Qualitative analysis

Qualitative data will be assessed using thematic analysis. Thematic analysis consists of five stages: familiarisation, generating codes, constructing themes, revising themes, and defining themes [43]. Draft codebooks for each participant group (e.g. mentor mothers, adolescents, and friends/family members) will be developed on an initial sub-set of transcripts by two researchers in each site. Following refinement based on researcher consensus, the final codebooks will be used to code each transcript independently by two researchers in each site. Sub-categories will be assessed for the number of occurrences across all transcripts and themes and categories relevant to the data identified. Findings will be triangulated with quantitative data on implementation, and mentor mother and adolescent outcomes to assess the feasibility and impact of the intervention.

### Feasibility criteria for progression to full trial

The primary objective of this study is to evaluate the feasibility and acceptability of the intervention, its implementation, and trial procedures for a subsequent large-scale evaluation. Overall feasibility and acceptability criteria for progression to a full trial have been informed by Mellor et al.’s [44] trial progression recommendations and retention criteria used in previous mental health research conducted in LMICs [45]. These consist of a mixture of qualitative and quantitative criteria:

- Identification of qualitative themes reporting that both adolescents and mentor mothers perceive the intervention and mentor training as being acceptable, feasible, and appropriate.
- Retention of at least 67% (i.e. 33% loss to follow-up) of adolescents and mentor mothers through endline assessments.
- Fewer than 15% missing items on outcome measures across all assessments.
- Presence of adverse events among fewer than 10% of participants.
- Identification of serious adverse events related to intervention delivery.

In domains where criteria are met, we will retain the procedure for the full trial. In domains where criteria are not met, we will modify procedures for the full trial guided by data collected during interviews. The presence of any adverse events and/or serious adverse events will be addressed by the research team to identify alternative strategies for the full trial and Data Safety Monitoring Committee. The number of feasibility, acceptability, and fidelity criteria that are not met will determine the extent of intervention and trial design modification.

### Identification of study limitation to inform a future trial

We will additionally monitor any issues related to recruitment, retention, assessments, and programme delivery throughout the study to identify and develop new strategies to improve our overall effectiveness and implementation science methodology. Local project co-ordinators will maintain constant dialog with the research team members who collect data, mentor mothers, and adolescent girls throughout the study. Information relevant to the conduct of the study will be shared with the project PI (TS) and Scientific Co-ordinator (KL).

## Discussion

Adolescent perinatal mental health has significant implications on the health of adolescents and their babies. However, few interventions have targeted the priorities, strengths, and needs for adolescent perinatal mental health in sub-Saharan Africa, where a large proportion of early pregnancies occur. The Thriving Mamas programme was co-designed with adolescent girls, their families, and communities to address this gap. Our understanding of the feasibility, acceptability, and appropriateness of the intervention beyond the perceptions of those involved in its development will allow for further refinement to its content and implementation strategies in preparation for testing its clinical impact. Additionally, the research will contribute to growing literature on the impact of co-design on the sustainability and success of mental health interventions.

Data collection is projected to begin in October 2023 and end in May 2024. As of September 2023, no participants have been recruited.

## Data Availability

Not applicable.

## Abbreviations

AIM: Acceptability of Intervention Measure
ENACT: Enhancing Assessment of Common Therapeutic Factors
FIM: Feasibility of Intervention Measure
GAD-7: Generalised Anxiety Disorder
IAM: Intervention Appropriateness Measure
INSPIRE: Innovative approaches to adolescent perinatal wellbeing
LMICs: Low- and middle-income countries
MICE: Multiple imputation using chained equations
MIDs: Clinical minimally important differences
MSPSS: Multidimensional Scale of Perceived Social Support
PHQ-9: Patient Health Questionnaire
SDS: Social Distance Scale
STAaRI: Standards for Reporting Implementation Studies
WHOQOL-BREF: World Health Organization Quality of Life brief version
